# Negative magnetoresistance in Dirac semimetal Cd_3_As_2_

**DOI:** 10.1038/ncomms10301

**Published:** 2016-01-08

**Authors:** Hui Li, Hongtao He, Hai-Zhou Lu, Huachen Zhang, Hongchao Liu, Rong Ma, Zhiyong Fan, Shun-Qing Shen, Jiannong Wang

**Affiliations:** 1 Department of Physics, The Hong Kong University of Science and Technology, Clear Water Bay, Hong Kong, China; 2 Department of Physics, South University of Science and Technology of China, Shenzhen, Guangdong 518055, China; 3 Department of Electronics and Computer Engineering, The Hong Kong University of Science and Technology, Clear Water Bay, Hong Kong, China; 4 Department of Physics, The University of Hong Kong, Pokfulam Road, Hong Kong, China

## Abstract

A large negative magnetoresistance (NMR) is anticipated in topological semimetals in parallel magnetic fields, demonstrating the chiral anomaly, a long-sought high-energy-physics effect, in solid-state systems. Recent experiments reveal that the Dirac semimetal Cd_3_As_2_ has the record-high mobility and positive linear magnetoresistance in perpendicular magnetic fields. However, the NMR has not yet been unveiled. Here we report the observation of NMR in Cd_3_As_2_ microribbons in parallel magnetic fields up to 66% at 50 K and visible at room temperatures. The NMR is sensitive to the angle between magnetic and electrical fields, robust against temperature and dependent on the carrier density. The large NMR results from low carrier densities in our Cd_3_As_2_ samples, ranging from 3.0 × 10^17^ cm^−3^ at 300 K to 2.2 × 10^16^ cm^−3^ below 50 K. We therefore attribute the observed NMR to the chiral anomaly. In perpendicular magnetic fields, a positive linear magnetoresistance up to 1,670% at 14 T and 2 K is also observed.

## Abstract (web summary)

Materials with topologically non-trivial band structures promise applications exploiting exotic electronic transport behaviour. Here, the authors observe a large negative magnetoresistance in Cd_3_As_2_ nanoribbons, evidence for a chiral transport anomaly required for its status as a Dirac semi metal.

## Graphical abstract

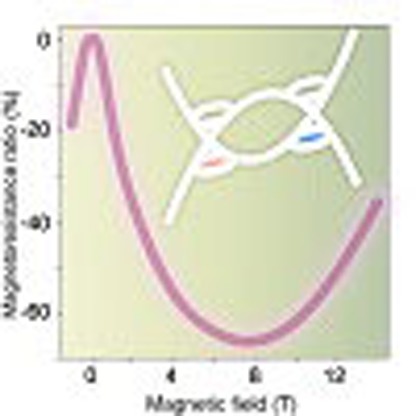

Searching for the signature of the Adler-Bell-Jackiw chiral anomaly^1^,^2^,^3^ in three-dimensional topological semimetals is one of the focuses in condensed matter physics. The topological semimetals have a band structure with the conduction and valence energy bands touching at a finite number of paired Weyl nodes^4^,^5^,^6^ (Fig. 1a). In each pair, the two Weyl nodes carry opposite chirality, and paired monopoles and anti-monopoles of Berry curvature in momentum space^7^ (Fig. 1b). The nontrivial Berry curvature can couple an external magnetic field (*B*) to the velocity of electrons, leading to a chiral current that is linearly proportional to the field. The correlation of chiral currents further contributes to an extra conductivity that quadratically grows with increasing magnetic field, in a magnetic field and an electric field applied parallel to each other. This *B*^2^-positive conductivity in weak parallel magnetic fields, or negative magnetoresistance (negative MR), is rare in non-ferromagnetic materials, thus can serve as one of the transport signatures of the topological semimetals. More importantly, because of its relation to the chiral charge pumping between paired Weyl nodes, the negative magnetoresistance is also believed to be a signature of the chiral anomaly^8^,^9^.

Among the recently identified candidates for topological semimetals (for example, HgCr_2_Se_4_ (refs 10, 11), (Bi_1−*x*_In_*x*_)_2_Se_3_ (ref. 12), Na_3_Bi (refs 13, 14, 15), TlBiSSe (ref. 16) and TaAs (refs 17, 18, 19, 20, 21)), the Dirac semimetal Cd_3_As_2_ (refs 22, 23, 24, 25, 26, 27) has peculiar transport properties, such as a giant MR in perpendicular magnetic fields and record-high mobility^28^,^29^,^30^,^31^,^32^,^33^, thus is of great potential in device applications. The negative MR possibly associated with the chiral anomaly has been claimed in several topological semimetals, including BiSb alloy^34^, ZrTe_5_ (ref. 35), TaAs (refs 36, 37), Na_3_Bi (ref. 38) and TaP (ref. 39). However, the chiral anomaly in Cd_3_As_2_ is not yet observed. One of the reasons is that the carrier density in earlier samples was too high (over 10^18^ cm^−3^). The chiral anomaly arises because of the nontrivial Berry curvature, which diverges at the Weyl nodes, so the Fermi energy *E*_F_ has to be as close to the Weyl nodes as possible for a clear signal of the negative MR.

In this work, we systematically investigate the magnetotransport properties of Cd_3_As_2_ microribbons, in which the carrier density is found to obey an Arrhenius's law, decreasing from 3.0 × 10^17^ cm^−3^ at 300 K to 2.2 × 10^16^ cm^−3^ below 50 K. In perpendicular magnetic fields, the ribbon exhibits a very large non-saturating positive linear MR (linear MR) up to 300 K. In contrast, when the magnetic field is applied in parallel with the measurement electric field, a negative MR is observed. It is sensitive to the angle between magnetic and electrical field, and shows a parabolic dependence on the low magnetic fields (<1 T) and persists up to 300 K. More importantly, our analysis reveals a characteristic carrier density dependence of the observed negative MR that is in agreement with the semiclassical theory about the chiral anomaly in topological semimetals. All the experimental evidence makes us believe that the chiral anomaly induced negative MR is indeed realized in our Cd_3_As_2_ ribbons with low carrier density. By studying the carrier density dependence of the observed linear MR in perpendicular magnetic fields, possible physical origins are also discussed. Our work shows that the carrier density plays an important role in the observation of the negative and linear MR and their magnitudes.

## Results

### Device characteristics

Figure 2a shows the scanning electron microscopy image of the four-terminal Cd_3_As_2_ devices studied in this work. The width *w* and inter-voltage-probe distance *l* are 1,210 and 1,600 nm, respectively. According to the atomic force microscopy measurement shown in Fig. 2b, the ribbon thickness *t* is about 327 nm (Fig. 2c). Figure 2d shows the measured temperature (*T*) dependence of the resistance (*R*) of the Cd_3_As_2_ ribbon. With decreasing temperature, the ribbon changes from an insulating behaviour to a metallic one, with a resistance peak appearing around 50 K. We note that similar *R*–*T* curves were also observed in recent studies of Dirac semimetals, where chiral anomaly induced transport features were reported^40^,^41^. One of the important properties of Dirac semimetals is the giant non-saturating linear magnetoresistance (linear MR) in high magnetic fields^28^,^29^,^30^,^31^,^32^,^33^. Indeed, our Cd_3_As_2_ ribbons do exhibit such an intriguing linear MR. Figure 2e shows the MR measured at *T*=2 K with varying angle between magnetic and electric field applied (see inset). When the magnetic field (*B*) is applied perpendicular to the ribbon in the *z* direction, that is, the *B*-field tilting angle *θ*=90^o^, a linear MR up to 1670% at 14 T is observed. This linear MR decreases at smaller titling angles when we rotate the *B*-field in the *x*–*z* plane. For *θ*≤10^o^, a negative MR begins to emerge in low magnetic fields, as shown in the Fig. 2f. For *θ*=0^o^, that is, the *B*-field is parallel to the electric field direction, the negative MR is the most prominent. We also rotate the *B*-field in the *x*–*y* plane and study the in-plane angle dependence of this negative MR (see Supplementary Note 1 and Supplementary Fig. 1 for details). The emergence of such a negative MR when *B*||*E* is recently ascribed to the chiral anomaly of topological semimetals^8^,^9^.

### Linear MR in perpendicular fields

To gain more physical insights into the observed linear MR and negative MR, we further study the temperature dependence of these two phenomena. Figure 3a shows the *R*–*B* curves measured at *θ*=90^o^ and different temperatures indicated. As temperature increases from 2 to 50 K, the linear MR in high magnetic fields (>8 T) is almost unchanged but it starts to weaken gradually as *T* further increases. At low temperatures, some wiggles are superimposed on the linear MR but quickly disappear as temperature increases. In addition, we note that the *R*–*B* curves show a quadratic *B*-field dependence in low magnetic fields (<1 T) distinct from the linear MR in high magnetic fields. As shown in the inset of Fig. 3b, the *R*–*B* curves at *T*=2 to 300 K can be well fitted by parabolas within a small *B*-field range. Such a parabolic MR is believed to arise from the Lorentz deflection of carriers and the *B*^*2*^ fitting allows us to deduce the carrier mobility, *μ*, by^42^


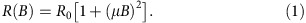


The obtained mobility of our Cd_3_As_2_ ribbon at different temperatures is shown in Fig. 3b. It increases monotonically with decreasing temperature. At *T*=2 K, the mobility reaches 10^4^ cm^2^V^−1^ s^−1^, which is comparable to those reported in previous studies of Cd_3_As_2_ (refs 29, 30, 32, 33). It has to be pointed out that equation (1) is valid when *B*<<1/*μ*. Considering the high mobility, we restrict the fitting range of the magnetic field below 0.15 T throughout the work. On the basis of Drude model of electric conduction, the carrier density *n* of our ribbon can be derived by


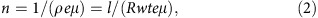


where *e* is the elementary charge, *ρ* is the resistivity of the ribbon and the resistance *R* at different temperatures is shown in Fig. 2c. Figure 3c shows the obtained carrier density as a function of temperature. Below 50 K, the carrier density is almost a constant. From 50 to 300 K, it increases with temperature by about one order of magnitude from 2.2 × 10^16^ to 3.0 × 10^17^ cm^−3^, following Arrhenius's law (solid curve in Fig. 3c):


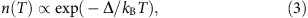


where *k*_B_ is the Boltzmann constant and the thermal activation energy Δ is about 51 meV. Such a thermally activated process of carriers accounts for the insulator-like *R*–*T* behaviour above 50 K shown in Fig. 2d, while the metallic behaviour below 50 K is mainly due to the increase of the carrier mobility with decreasing temperature. Since the intrinsic carriers will not follow Arrhenius's law for Dirac fermions^43^, we tentatively ascribe the thermal activation of carriers to some traps present in our Cd_3_As_2_ ribbons. It is worth pointing out that both the observed linear MR and the carrier density are almost temperature independent when *T*<50 K and become temperature dependent when *T*>50 K. This coincidence implies that the observed strength of the linear MR is related to the change of carrier density. We can thus examine the temperature dependence of the linear MR in the temperature range from 50 to 300 K in terms of the carrier density. The slope *k* of the linear MR is extracted by linearly fitting the *R*–*B* curves between 8 and 14 T, as shown in Fig. 3a. The obtained *k* as a function of *n* is shown in Fig. 3d.

A giant non-saturating linear MR has been observed in various Dirac semimetals^28^,^29^,^30^,^31^,^32^,^33^, but its physical origin is still under debate. One possible mechanism is the quantum linear MR model proposed by Abrikosov^44^, where a non-saturating linear MR would appear in three-dimensional gapless semiconductors with linear energy dispersion when all the carriers are condensed into the lowest bands of Landau levels, that is, in the quantum limit. According to this model, the linear MR is temperature independent but should follow the 1/*n*^2^ dependence, that is, 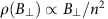, where *n*^2^ arises because the longitudinal resistance 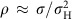 in the limit that the longitudinal conductivity *σ* is much smaller than the Hall conductivity *σ*_H_ and the Hall conductivity is proportional to *n*. Abrikosov's model with linear dispersion leads to a longitudinal *σ* independent on the carrier density, which may be violated in real materials. If *σ* is also proportional to *n*, as in most cases, the carrier density dependence should be corrected to 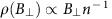. As a result, the slope of the quantum linear MR is inversely proportional to the carrier density. For comparison we plot *k*∝*n*^−1^ (red solid line) and *k*∝*n*^−2^ (blue solid line) curves in Fig. 3d. As it can be seen, the measured *k* follows *n*^−1^ dependence. In addition, our system is believed to enter the quantum limit in the fitting magnetic field range from 8 to 14 T, as will be discussed later in this work. All these seem to suggest the quantum model as the underlying physical origin of the observed linear MR in high fields shown in Fig. 3a. Besides this quantum model, there is another classical model proposed by Parish and Littlewood^45^ to account for the linear MR observed in polycrystalline silver chalcogenides. It is the disorder-induced admixture of the Hall signal that gives rise to the linear MR. Considering the low carrier density and quite high mobility of our Cd_3_As_2_ ribbons, this classical model is unlikely applicable to the observed linear MR. Also, there is explanation in the presence of balanced electron and hole carriers^46^, which is apparently not our case. The linear transverse MR observed in different systems may have various origins, and is still a challenging theoretical question^47^,^48^.

### Negative MR in parallel fields

**Figure 1:**
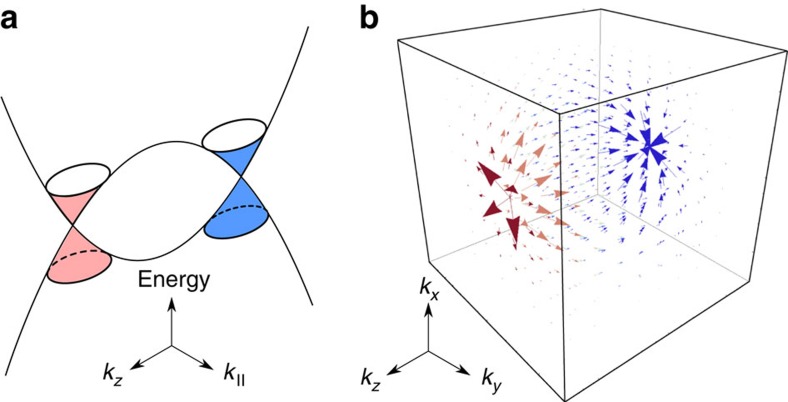
Nontrivial band structure and Berry curvature of a topological semimetal. (**a**) A schematic of the energy spectrum of a topological semimetal. (*k*_*x*_, *k*_*y*_, *k*_*z*_) is the wave vector. 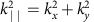 . (**b**) The vector plot of the Berry curvature in momentum space. The conduction and valence bands of a topological semimetal touch at the Weyl nodes, at which a pair of monopoles is hosted. The arrows show that the flux of the Berry curvature flows from one monopole (red) to the other (blue), defining the nontrivial topological properties of a topological semimetal.

**Figure 2:**
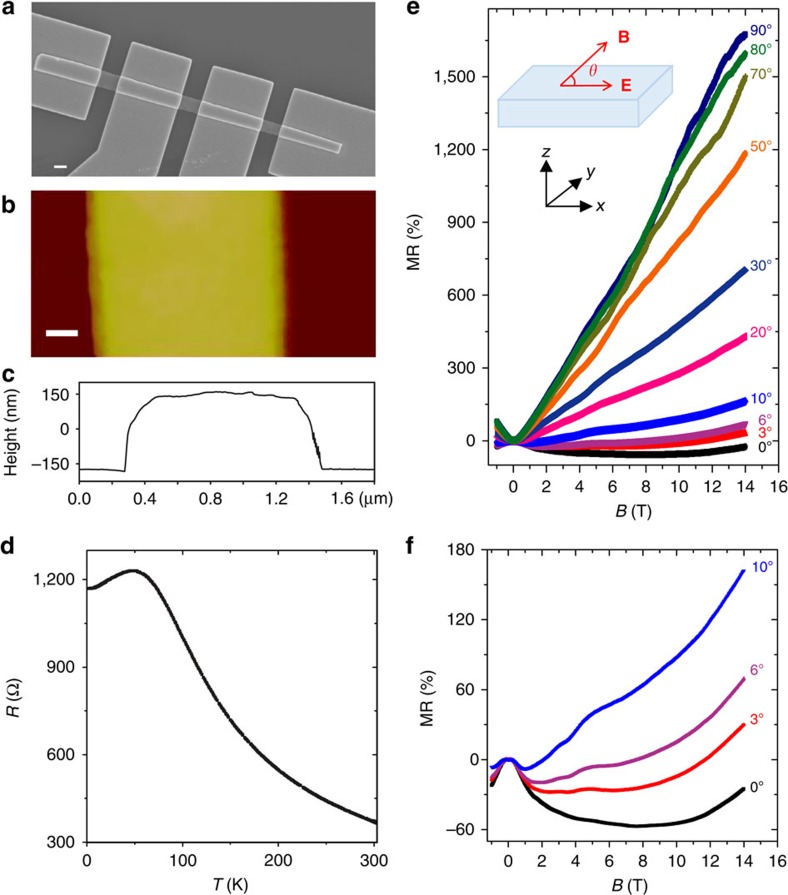
Topological semimetal Cd_3_As_2_ microribbon device and magnetotransport characteristics. (**a**) The scanning electron microscopy and (**b**) atomic force microscopy images of the device. Scale bars, 1 μm (**a**); 200 nm (**b**). (**c**) The height profile of the Cd_3_As_2_ microribbon in **b**. (**d**)The measured resistance (*R*) as a function of temperature (*T*) at zero magnetic field. (**e**) The magnetoresistance (MR) measured at 2 K with applied magnetic field (*B*) direction changing from perpendicular (*θ*=90°) to parallel (*θ*=0°) to the electric field (*E*) direction in the *z–x* plane. (**f**) The replot of MR with *θ*<10° showing the negative MR at low magnetic fields.

In Fig. 4a, the *R*–*B* curves obtained at *θ*=0^o^ are shown at different temperatures. There exists a critical *B*-field for each curve, below which a pronounced negative MR is observed even with temperature up to 300 K. At *T*=50 K and *B*=8 T, the highest negative MR of 66% can be obtained. It is also noted in Fig. 4a that some MR ripples are superimposed on the negative MR especially as low temperatures. Besides the apparent negative MR, a small resistance dip appears in low fields with *T*<20 K, as shown in Fig. 4b. Such a dip is believed to arise from the weak anti-localization effect in Dirac semimetals^34^,^49^. In Fig. 4c, the low-field-negative MR is shown at different temperatures. Note that the measured resistance has been converted to the conductivity *σ* and Δ*σ*=*σ*−*σ*_0_, where *σ*_0_ is the conductivity at zero magnetic field. Remarkably, all the data in Fig. 4c show a quadratic dependence on the magnetic field, as indicated by the fitting curves with Δσ=*C*_a_*B*^2^. This is consistent with the prediction in previous theoretical studies^8^,^9^. When an external electric field is applied in parallel with the magnetic field, chiral charges will be pumped from one Weyl node to the other, as a result of the nontrivial Berry curvature (see Fig. 1b). The chiral charge is therefore not conserved at each Weyl node. Such a chiral anomaly is expected to give rise to a prominent positive conductivity proportional to *B*^2^ based on a semiclassical transport calculation^8^,^9^. It should be noted that such a quadratic field dependence of the conductivity is only valid in weak magnetic fields or high temperatures such that the Landau quantization can be ignored. Indeed, the quadratic field dependence of Δ*σ* in Fig. 4c occurs in low magnetic fields (<0.8 T).

**Figure 3:**
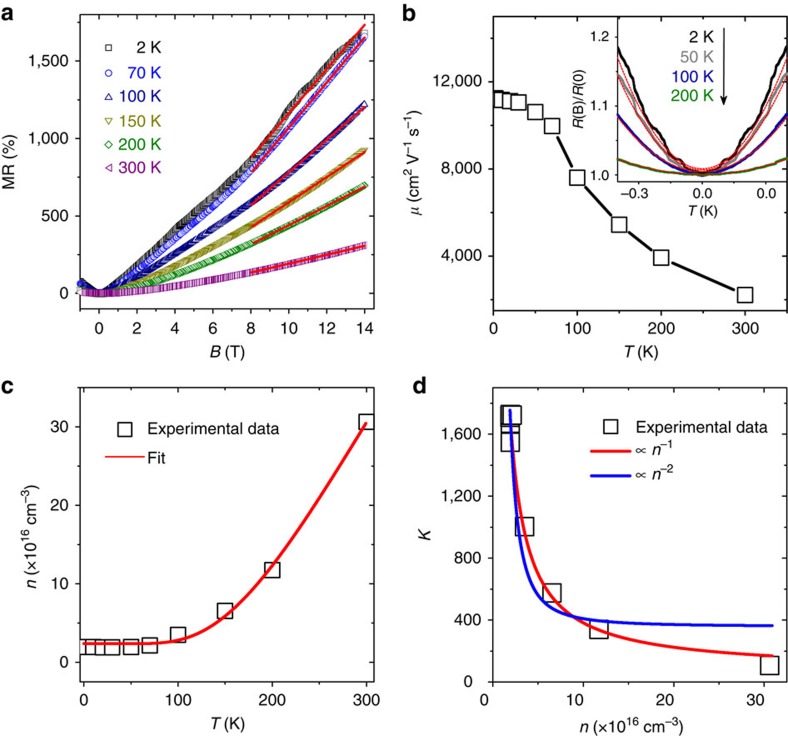
Linear MR in *B* perpendicular to *E*. (**a**) The MR measured at different *T* indicated. (**b**) The *T* dependence of the carrier mobility, which is calculated using Kohler's rule or equation (1). Inset: the MR at small magnetic fields and the parabola fittings (solid lines) at different *T* indicated. (**c**) The carrier density as a function of *T*. The solid red curve is the fitting using Arrhenius's law. (**d**) The slope *k* of the linear MR at high *B* (from 8 to 14 T, obtained in **a**, see solid curves) as a function of carrier density, *n*. The solid red and blue curves are the fittings using *n*^−1^ and *n*^−2^, respectively. The corresponding temperature for each data point is also indicated.

**Figure 4:**
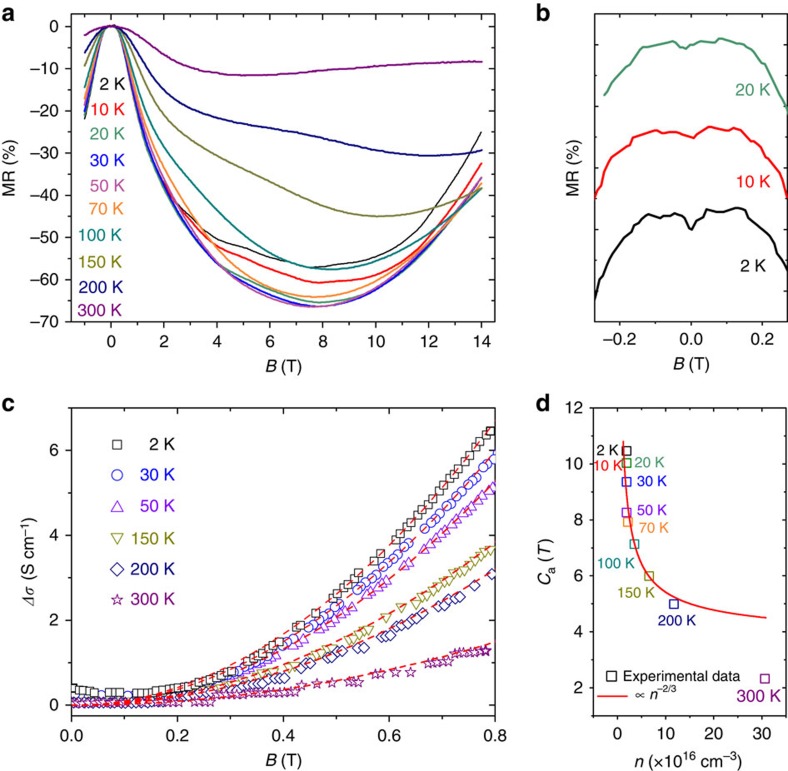
Negative MR in *B* parallel to *E*. (**a**) The MR measured at different *T* indicated. (**b**) The weak anti-localization effect at low temperatures and very small magnetic fields. (**c**) The positive conductivity Δ*σ*=*σ*–*σ*_0_, where σ_0_ is the conductivity at *B*=0, converted from measured negative MR at different *T* indicated. The solid red curves are the fittings using Δ*σ*=*C*_a_*B*^2^. (d) *C*_a_ as a function of the carrier density, *n*. The solid red curve is a fitting using *n*^−2/3^. The corresponding temperature for each data point is also indicated.

Furthermore, because the chiral anomaly arises from the nontrivial Berry curvature, which diverges at the Weyl nodes, the positive conductivity will increase with decreasing Fermi energy and carrier density. More specifically, the theory predicts 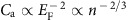 if the Fermi level *E*_F_ is close to the Weyl nodes^8^,^9^. As a result, we plot the obtained fitting parameter *C*_a_ as a function of the carrier density in Fig. 4d together with a 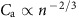 curve (solid red line). One can see that *C*_a_ does obey the *n*^−2/3^ relationship reasonably well. All these experimental evidences, that is, measured Δ*σ*=*C*_a_*B*^2^ and 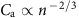, lead us to believe that it is the chiral anomaly that gives rise to the observed negative MR shown in Fig. 4a. In the above discussion, we only consider the influence of *n* on *C*_a_. But *C*_a_ is also proportional to the internode scattering time *τ*_a_ (refs 8, 9). Since the momentum transfer assisted by phonons is suppressed at low temperatures, *τ*_a_ will increase with decreasing temperature. As shown in Fig. 3c, the carrier density of our sample follows Arrhenius's law. *τ*_a_ is thus expected to increase with decreasing *n*. At higher carrier density, the deviation from perfect linear dispersion can also lead to a correction to the power law of *n*. Therefore, the *n*^−2/3^ fitting of *C*_a_ in Fig. 4d, which assumes a constant *τ*_a_ for different *n*, would over- and underestimate the value of *C*_a_ at high and low carrier densities, respectively. This accounts for the deviation of *C*_a_ from the fitting at 300 K and below 50 K shown in Fig. 4d. The observation of chiral anomaly induced negative MR also requires *τ*_a_≫*τ*, where *τ* is the momentum relaxation time. In Supplementary Note 2 and Supplementary Fig. 2, the ratio of *τ*_a_/*τ* has been estimated. *τ*_a_ is at least one order of magnitude larger than *τ*, in consistent with the theories^8^,^9^.

It is well known that, in solids, negative MR may have other physical origins. It can arise from the weak localization effect due to the quantum interference of time-reversed scattering loops. Since the phase coherence length decreases rapidly with increasing temperature, the weak localization can only be observed at low temperatures. The negative MR observed in Cd_3_As_2_ ribbons persists up to 300 K; therefore, cannot be attributed to the weak localization effect. Magnetic scattering might be another mechanism for negative MR, as reported in some magnetic systems^50^, but our Cd_3_As_2_ ribbons are non-magnetic.

### MR in the quantum limit

**Figure 5:**
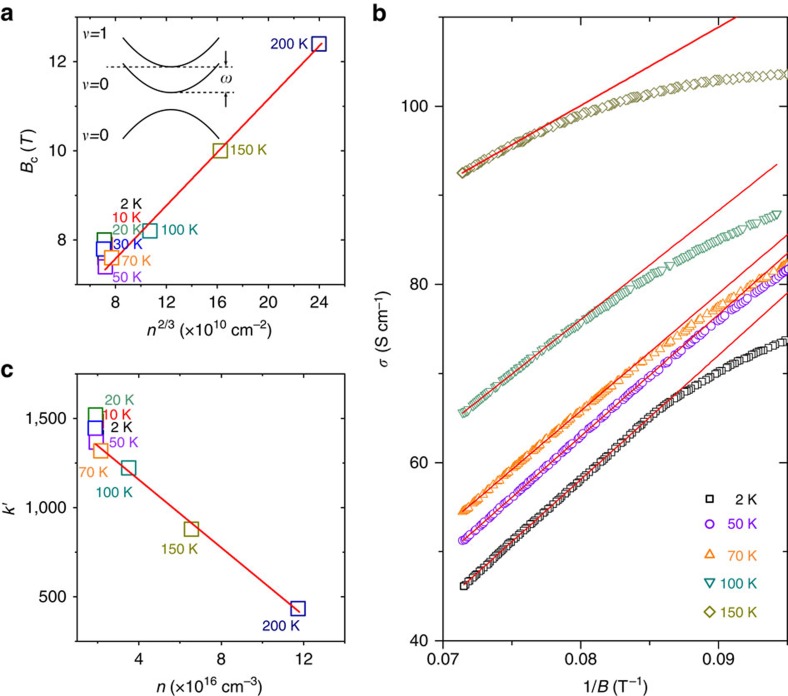
MR in the quantum limit. (**a**) *B*_C_ as a function of *n*^*2/3*^, *n* is the carrier density. The corresponding temperature for each data point is also indicated. The solid red line is a linear fitting. *B*_C_ is defined as the critical field at which the MR in *B* parallel to *E* is minimum. At *B*_C_, the system enters the quantum limit with a structure of the Landau bands illustrated as in the inset. *v* is the index of the Landau bands and *ω* is the cyclotron frequency. (**b**) The high-*B*-field magnetoconductivity in *B* parallel to *E* as a function of 1/*B*. The magnetoconductivity is found to follow a *k*′/*B* dependence while approaching 14 T (see solid red lines). (**c**) The slope *k*′ as a function of the carrier density. The solid red line is a linear fitting.

As mentioned above, a critical magnetic field *B*_C_ exists for each MR curve to characterize the change of MR from negative to positive in Fig. 4a, as illustrated by the arrows. We believe that this sign change in MR is an indication of the system to enter the quantum limit at *B*_C_, that is, the Fermi energy crosses only the lowest Landau bands with the band index *v*=0 and lies right below the bottom of the *v*=1 Landau bands, as shown in the inset of Fig. 5a. The energy spacing between the *v*=1 and *v*=0 bands is roughly related to the cyclotron frequency as 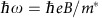, with m* as the effective mass of carriers in Cd_3_As_2_. On the other hand, 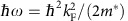, where the Fermi wave vector in the *v*=0 bands is related to the carrier density according to 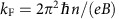. Note that there is no spin degeneracy for the lowest Landau band of Dirac semimetal (Supplementary Note 3; Supplementary Fig. 3). Combing them, we can deduce a relationship between the critical field and the carrier density as 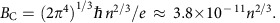 To justify this scenario, we extract *B*_C_ from the MR curves at different temperatures (except 300 K curve) in Fig. 4a, and plot them in terms of the carrier density *n*^2/3^ as it is shown in Fig. 5a. As the carrier density only changes within the temperature range of 50–200 K, a straight line fitting of the data in this range with *B*_C_=*βn*^2/3^ yields *β*=3.0 × 10^−11^, which is close to the above theoretical value. Such a good agreement strongly supports our assumption that our system is indeed in the quantum limit above *B*_C_. The deviation of data below 50 K, where the carrier density is almost constant, is probably caused by the superimposed ripples on the MR that prevent an accurate extraction of *B*_C_. As it is shown in Fig. 4a, after the system is in the quantum limit, the measured magnetoresistance becomes positive, or the magnetoconductivity becomes negative. We find at high fields approaching 14 T, the magnetoconductivity follows a good *B*^−1^ dependence, as indicated by the linear fittings in Fig. 5b (red solid curves). We also find that the obtained slope 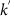 of these linear fittings is inversely proportional to the carrier density *n* (see Fig. 5c). This observed negative linear magnetoconductivity at high fields is contrary to the theoretical expectation, in which a positive linear magnetoconductivity is predicted as an additional signature of the chiral anomaly^8^,^51^,^52^. However, in reality, the relaxation time and Fermi velocity can bring extra magnetic field dependences, leading to either positive or negative magnetoconductivity in Dirac semimetals^53^.

## Discussion

In conclusion, we have performed a systematic magnetotransport study of Cd_3_As_2_ microribbons. Due to the low carrier density in our samples, we can observe both the non-saturating linear MR in a high perpendicular magnetic field and the quadratic negative MR when a weak magnetic field is in parallel with the measurement electric field. Furthermore, the thermally activated behaviour of carriers in our Cd_3_As_2_ ribbons allows us to study the carrier density dependence of these two phenomena. Although the mechanism for the linear MR is still not well understood, the quadratic negative MR can be safely ascribed to the chiral anomaly intrinsic to the Dirac semimetal Cd_3_As_2_. Our work provides new physical insights into the intriguing transport properties of Dirac semimetals, revealing the importance of carrier density in mediating the linear MR in perpendicular fields and the quadratic negative MR in parallel fields. It also calls for the thin-film growth of Dirac semimetals. By applying an external gate to effectively tune the Fermi level of the film towards the Weyl nodes, much larger linear MR and prominent negative MR would be expected.

## Methods

### Growth

The Cd_3_As_2_ microribbon was grown by a chemical vapour deposition method. Cd_3_As_2_ powders and Si (001) covered with 2-nm Au layer were used as the precursor and substrates, respectively, and argon were used as a carrier gas. Before each growth, the furnace was pumped and flushed for several times to remove water and oxygen using dry argon. The precursor powder boat was placed in the hot centre of the furnace, while the Si (100) substrates were placed downstream about 32 cm away from the precursor powders. The furnace was gradually heated up to 750 °C in 40 min, and the Ar flow was kept as 100 s.c.c.m. during the growth process. The typical growth time is 60 min, and after then the furnace was cooled down to room temperature naturally.

### Characterization

Structural investigations have been performed on scanning electron microscopy (JEOL-6300) and atomic force microscopy (Dimension 3100) at room temperature. The growth direction of the Cd_3_As_2_ microribbon has been confirmed by the transmission electron microscopy (JEM 2010, JEOL) measurements. The high-resolution transmission electron microscopy identified [110] as the growth or axial direction of the ribbon (Supplementary Note 4 and Supplementary Fig. 4).

### Devices fabrication and magnetoresistance measurements

To study the magnetotransport properties, a four-terminal device was fabricated with standard electron-beam lithography and lift-off processes. Au/Cr electrodes with the thickness of 275/25 nm were deposited using thermal evaporation methods. The transport properties of the device were then investigated in a Quantum Design PPMS system with the highest magnetic field up to 14 T.

## Additional information

**How to cite this article:** Li, H. *et al*. Negative magnetoresistance in Dirac semimetal Cd_3_As_2_. *Nat. Commun.* 7:10301 doi: 10.1038/ncomms10301 (2016).

## Supplementary Material

Supplementary Information Supplementary Figures 1-4, Supplementary Notes 1-4, and Supplementary References
