# Supplementary material for: Negative magnetoresistance in Dirac semimetal Cd3As2
Source: Nat Commun. 2016 Jan 8;7:10301. doi: 10.1038/ncomms10301 (PMC4729874; doi:10.1038/ncomms10301)
Supplement: Supplementary Information — Supplementary Figures 1-4, Supplementary Notes 1-4, and Supplementary References [file ncomms10301-s1.pdf]

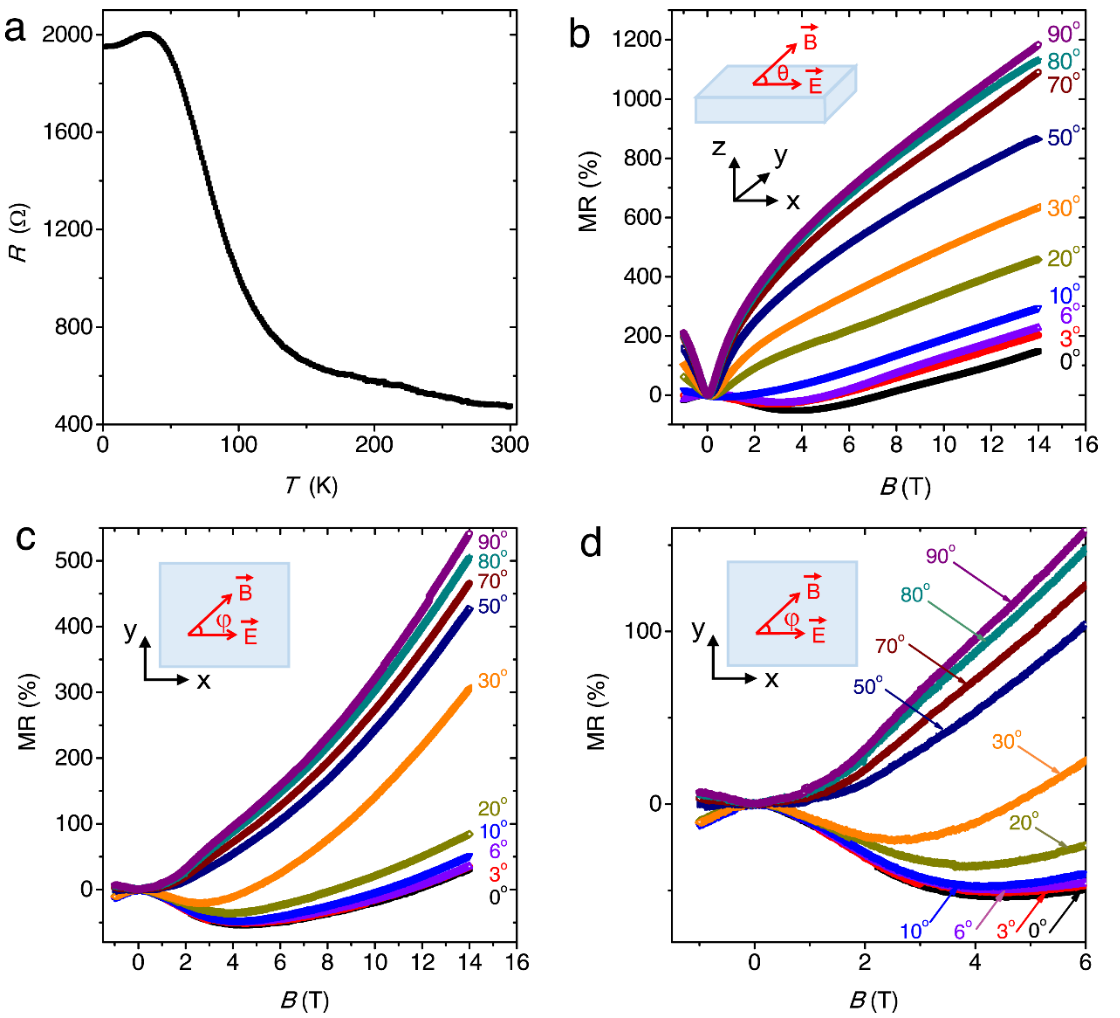

Supplementary Figure 1. Magneto-transport characteristics of topological semimetal Cd_3As_2 microribbon. (**a**) Measured resistance (*R*) as a function of temperature (*T*) at zero magnetic field. (**b**) Magnetoresistance (MR) measured at 2 K with applied magnetic field (*B*) direction changing from perpendicular $\theta = 90^0$ to parallel $\theta = 0^0$ to the electric field (*E*) direction in *z*-*x* plane. (**c**) Magnetoresistance (MR) measured at 2 K with applied magnetic field (*B*) direction changing from perpendicular $\varphi = 90^0$ to parallel $\varphi = 0$ to the electric field (*E*) direction in *y*-*x* plane. (**d**) Replot of MR curves of (c) showing the negative MR at $\varphi < 50^0$.

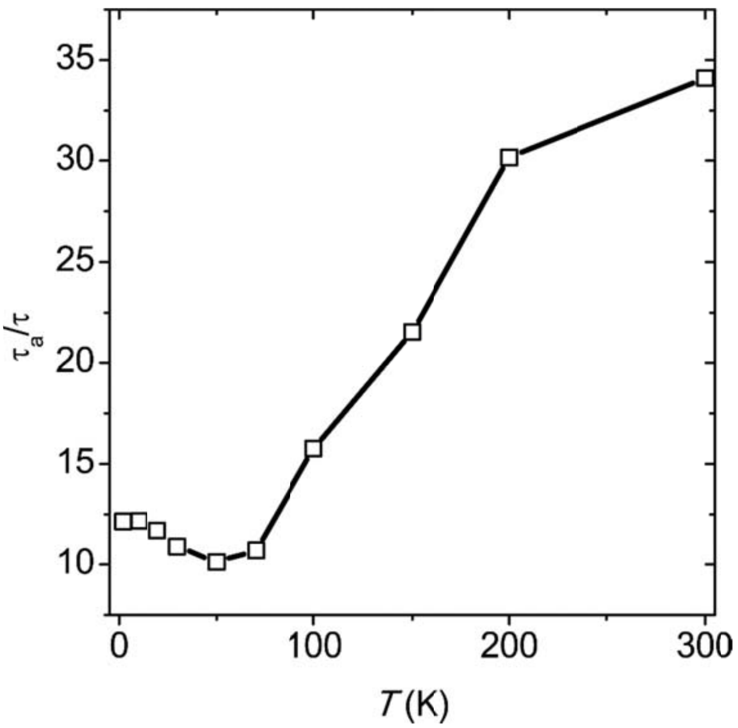

Supplementary Figure 2. Relaxation time of topological semimetal Cd_3As_2 microribbon. Temperature dependence of the ratio of the axial relaxation time τ_a and momentum relaxation time τ for Cd_3As_2 microribbon.

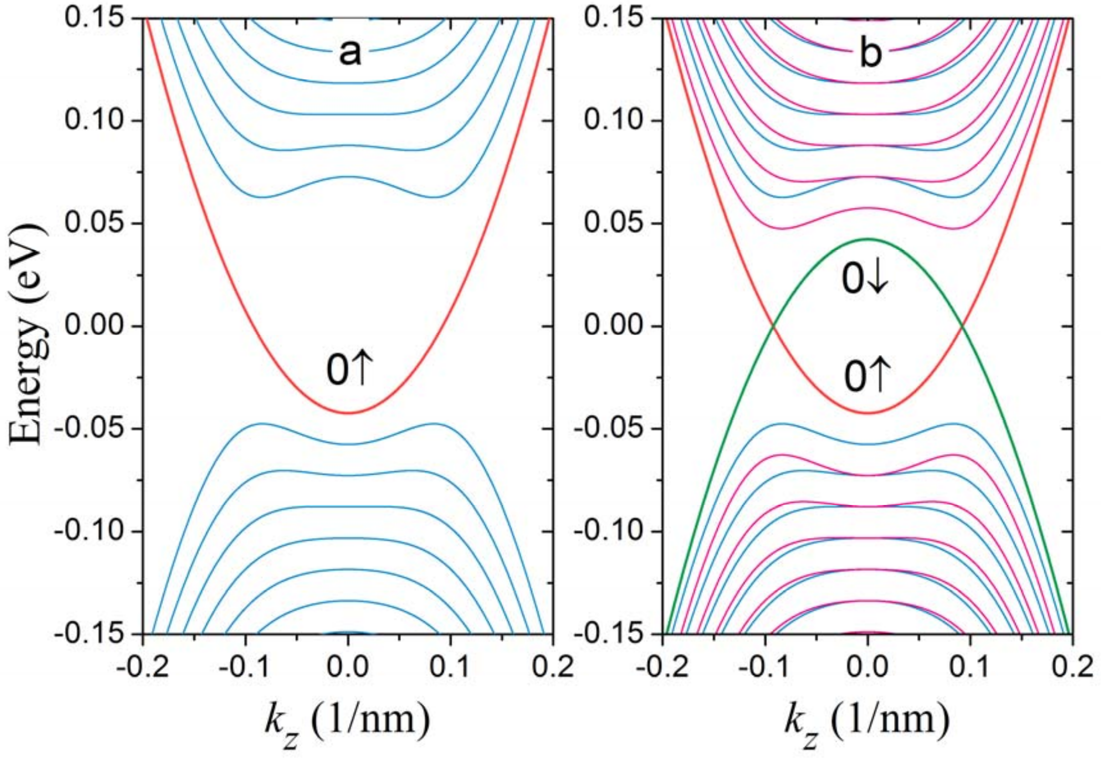

Supplementary Figure 3. The Landau bands in the z-direction magnetic field *B*. (**a**) The Landau bands for a Weyl semimetal. (**b**) The Landau bands in a Dirac semimetal.

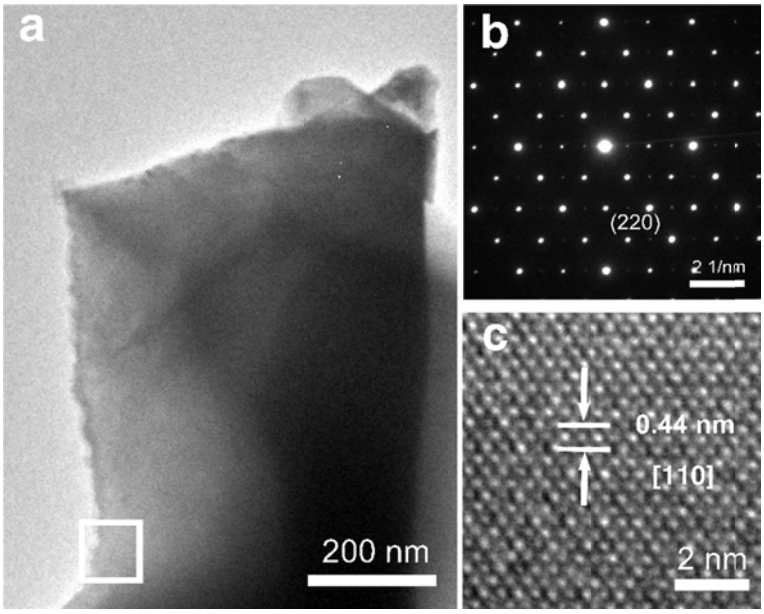

Supplementary Figure 4. Structural characteristics of Cd_3As_2 microribbon. (**a**) The TEM image of the Cd_3As_2 ribbon microstructures. (**b**-**c**) The SAED pattern and HRTEM image of the ribbon collected in the marked area in (a).

Supplementary Note 1: Magneto-transport characteristics of topological semimetal Cd_3As_2 microribbon

Supplementary Figure 1(a) shows the measured temperature (*T*) dependence of the resistance (*R*) of another Cd_3As_2 ribbon devices. Similar to the devices measured in the manuscript, with decreasing temperature, the resistance increase, and reach the maximum at the critical temperature around 34 K, corresponding to the ribbon changes from an insulating behavior to a metallic one. The magnetoresistance (MR) were measured at T = 2 K with varying angle between magetic and electric field applied in *z*-*x* plane (see Supplementary Fig. 1(b)). When the magnetic field (*B*) is applied perpendicular to the ribbon in the *z* direction, *i.e.* the *B*-field tilting angle θ = 90°, a linear MR up to 1180% at 14 T is observed. A negative MR begins to emerge in low magnetic fields when $\theta \leq 10^o$, and the negative MR is the most prominent when *B*-field is parallel to the electric field direction, *i.e.* $\theta = 0^o$. The largest negative MR of -53% appearing at 3.8 T is observed.

The in-plane angle dependence of MR was also measured, as shown in Supplementary Figure 1(c). When the *B*-field is parallel to the electric field in *x*-*y* plane (φ=0°), negative MR was observed. This negative MR decreases with increasing angles and finally vanishes around φ=50° (see in the Supplementary Figure 1(d)). When the magnetic field is applied perpendicular to the ribbon in the *y* direction ($\varphi = 90^o$), a positive MR up to 540% at 14 T is observed. We notice that our in-plane results are very similar to those of another Dirac semimetal Na_3Bi, where chiral anomaly induced negative MR was reported recently[1]. Therefore, the negative MR observed in our Cd_3As_2 ribbons is really related to the chiral anomaly of Dirac semimetals.

Supplementary Note 2: Estimate of the axial relaxation time τ_a and momentum relaxation time τ

Based on the formula given by Burkov[2] for a pair of Weyl nodes, we find the coefficient in front of the chiral anomaly term of the magnetoconductivity as

$$C_a = 2 \times \frac{e^4 \tau_a}{4\pi^4 \hbar^4 g(E_F)},$$

where in SI units we have recovered the missing $\hbar^4$, "2 ×" is due to two pairs of Weyl nodes (*i.e.*, 4 Weyl nodes) in the Dirac semimetal, $g(E_F)$ is the density of states of two Weyl nodes and

$$g(E_F) = \frac{E_F^2}{\pi^2 \hbar^3 v_x v_y v_z},$$

where v_x, v_y, and v_z are the velocities along three directions, and in SI units we have recovered the missing $\hbar^3$. According to Liu et al,[3] $v_x = 1.28 \times 10^6$ m/s, $v_y = 1.30 \times 10^6$ m/s, $v_z = 0.327 \times 10^6$ m/s.

The Fermi energy E_F can be found from the carrier density n,

$$n = \frac{4\pi}{3} \frac{E_F^3}{(2\pi)^2 \hbar^3 v_x v_y v_z}.$$

The momentum relaxation time τ can be found from the conductivity at zero magnetic field[5]

$$\sigma(0) = 2e^2 g(E_F) \frac{v_x^2}{3} \tau \frac{3}{2},$$

where 2 arises because we have two pairs of Weyl nodes, 3/2 is the vertex correction to the velocity.

Combing the above formulas, we arrive at

$$\tau_a = \frac{2\pi^2 \hbar E_F^2 C_a}{e^4 v_x v_y v_z}, \quad \tau = \frac{\sigma(0)\pi^2 \hbar^3 v_y v_z}{e^2 E_F^2 v_x}, \quad \text{and} \quad \frac{\tau_a}{\tau} = \frac{E_F^4 C_a}{e^2 \sigma(0) \hbar^2 v_y^2 v_z^2}.$$

As shown in Supplementary Figure 2, the axial relaxation time τ_a is one order larger than that of the momentum relaxation time τ in the whole temperature region. The

large ratio τ_a/τ demonstrates the existence of the axial relaxation process between the paired Weyl nodes, and the charges pumped between paired nodes may result in the NMR, which is the signature of the chiral anomaly.

Supplementary Note 3: Non-degeneracy Weyl cones of topological semimetal Cd_3As_2

Unlike in conventional metals, the lowest Landau bands in the Dirac semimetal Cd_3As_2 have no spin degeneracy. In the absence of the magnetic field, the band structure of Cd_3As_2 is two-fold degenerate because of time reversal symmetry, and is formed by a Weyl semimetal and its time-reversal counterpart. We have taken this degeneracy into account in our calculation. In the magnetic field, the lowest Landau bands from the Weyl semimetal and its time-reversal counterpart must have opposite velocity (chirality), so its lowest Landau bands look like that in Supplementary Figure 3(b)[4], where the Landau band calculation is based on a model constructed from the first-principle calculations[5]. The red curve ($0\uparrow$) is the lowest Landau band from the Weyl semimetal and the green one ($0\downarrow$) is from its time-reversal counterpart. Please note that the separation of two Weyl nodes is finite in the case. At the critical field where the system enters the quantum limit, the red and green bands may be separated, or not separated as shown in Supplementary Figure 3(b). In either case, the calculation of the critical field does not have to include the spin degeneracy because of the unique chiral property of the topological semimetal.

Supplementary Note 4: Structure Characterization of Cd_3As_2 microribbon

Supplementary Fig. 4(a) shows the transmission electron microscopy (TEM) image of a Cd_3As_2 microribbon. The width of the ribbon is about 500 nm. Selected area electron diffraction (SAED) pattern measured in the maked area in Supplementary Fig. 4(a) [see Supplementary Fig. 4(b)] shows the single crystalline character of the Cd_3As_2 ribbon. The average *d*-spacing of 0.44 nm measured in high resolution TEM image [see Supplementary Fig. 4(c)] corresponding to the lattice spacing of (110) plane of Cd_3As_2, indicating the growth or axial direction of the Cd_3As_2 ribbon is [110], and is consistent with the SAED pattern. When the electrodes were deposited along the longitudinal direction of the Cd_3As_2 ribbon, and the current is flow along the [110] direction of ribbon during the whole transport measurements. A similar current direction in Cd_3As_2 along [1-10] has been reported recently[6].

Supplementary References

1. Xiong, J. et al. Evidence for the chiral anomaly in the Dirac semimetal Na_3Bi. *Science* **10,** 1126 (2015).
2. Burkov, A. A. Chiral anomaly and diffusive magnetotransport in Weyl metals. *Phys. Rev. Lett.* **113,** 247203 (2014).
3. Liu, Z. K. et al. A stable three-dimensional topological Dirac semimetal Cd_3As_2. *Nature Mater.* **13,** 677–681 (2014).
4. Lu, H.-Z., Zhang, S.-B. & Shen, S.-Q. High-field magnetoconductivity of topological semimetals with short-range potential. *Phys. Rev. B* **92**, 045203 (2015).
5. Wang, Z., Weng, H., Wu, Q., Dai, X. & Fang, Z. Three-dimensional Dirac semimetal and quantum transport in Cd_3As_2. *Phys. Rev. B* **88,** 125427 (2013).
6. Zhao, Y. F. et al. Anisotropic fermi surface and quantum limit transport in high mobility three-dimensional Dirac semimetal Cd_3As_2. *Phys. Rev. X* **5,** 031037 (2015).
